# Supplementary material for: Mutations in PCYT2 disrupt etherlipid biosynthesis and cause a complex hereditary spastic paraplegia
Source: Brain. 2019 Oct 22;142(11):3382–97. doi: 10.1093/brain/awz291 (PMC6821184; doi:10.1093/brain/awz291)
Supplement: awz291_Supplementary_Data [file awz291_supplementary_data.zip › awz291-Suppl_data/Supplementary_Data4.zip!/Supplementary information 4 - plasma lipidomics.zip!/Plasma lipidomics/Control vs Patient3b/Statistical plots and data/Heatmap sorted on p-value and FC.pdf]

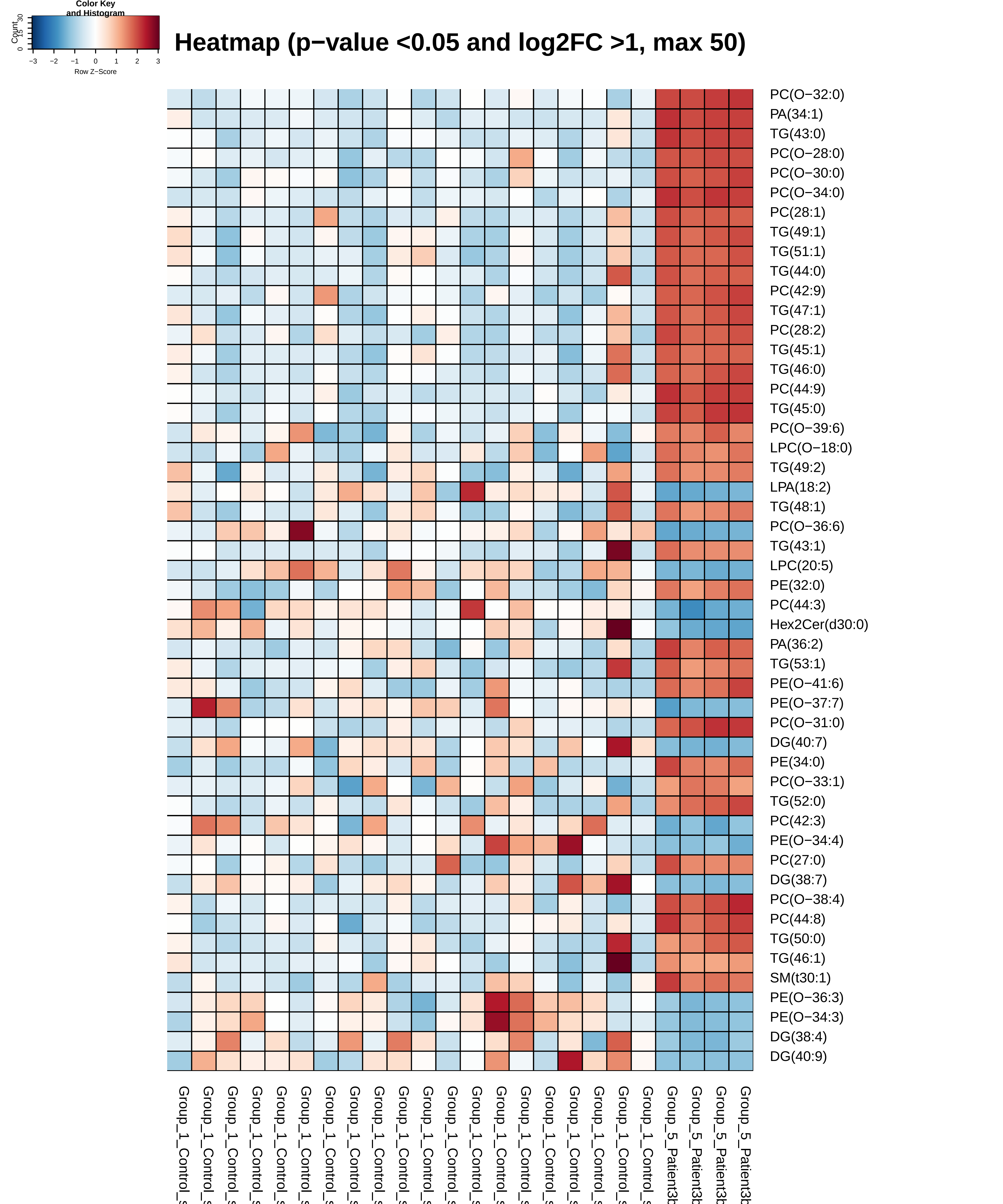
Color Key
and Histogram
Count
0
15
30
−3
−2
−1
0
1
2
3
Row Z−Score
Heatmap (p−value <0.05 and log2FC >1, max 50)
PC(O−32:0)
PA(34:1)
TG(43:0)
PC(O−28:0)
PC(O−30:0)
PC(O−34:0)
PC(28:1)
TG(49:1)
TG(51:1)
TG(44:0)
PC(42:9)
TG(47:1)
PC(28:2)
TG(45:1)
TG(46:0)
PC(44:9)
TG(45:0)
PC(O−39:6)
LPC(O−18:0)
TG(49:2)
LPA(18:2)
TG(48:1)
PC(O−36:6)
TG(43:1)
LPC(20:5)
PE(32:0)
PC(44:3)
Hex2Cer(d30:0)
PA(36:2)
TG(53:1)
PE(O−41:6)
PE(O−37:7)
PC(O−31:0)
DG(40:7)
PE(34:0)
PC(O−33:1)
TG(52:0)
PC(42:3)
PE(O−34:4)
PC(27:0)
DG(38:7)
PC(O−38:4)
PC(44:8)
TG(50:0)
TG(46:1)
SM(t30:1)
PE(O−36:3)
PE(O−34:3)
DG(38:4)
DG(40:9)
Group_1_Control_s
Group_1_Control_s
Group_1_Control_s
Group_1_Control_s
Group_1_Control_s
Group_1_Control_s
Group_1_Control_s
Group_1_Control_s
Group_1_Control_s
Group_1_Control_s
Group_1_Control_s
Group_1_Control_s
Group_1_Control_s
Group_1_Control_s
Group_1_Control_s
Group_1_Control_s
Group_1_Control_s
Group_1_Control_s
Group_1_Control_s
Group_1_Control_s
Group_5_Patient3b
Group_5_Patient3b
Group_5_Patient3b
Group_5_Patient3b
